# The Impact of Mobile Health Literacy, Socioeconomic Factors, and Engagement Patterns on DiabetesXcel App Usage in Adults

**DOI:** 10.1007/s40200-025-01725-2

**Published:** 2025-09-25

**Authors:** Jainam Shah, Scott Wilson, Kevin Wang, Sherron Thomas, Sachin Pathuri, Jason Zheng, Ibrahim Furkan Acar, Eleni Kohilakis, Sanjana Boyapalli, Vivian Kim, Ashley Berlot, Wenzhu Mowrey, Savneet Kaur, Lakshmi Priyanka Mahali, Jeffrey Gonzalez, Judith Wylie-Rosett, Sunit P. Jariwala

**Affiliations:** 1 Albert Einstein College of Medicine, 1300 Morris Park Avenue, Bronx, NY 10461 United States of America; 2 Department of Medicine, Brigham & Women’s Hospital, Boston, MA United States of America; 3 Department of Internal Medicine, Beth Israel Deaconess Medical Center, 330 Brookline Avenue, Boston, MA 02215 United States of America; 4 Department of Epidemiology & Population Health, Albert Einstein College of Medicine, 1300 Morris Park Avenue, Bronx, NY 10461 United States of America; 5 Montefiore Medical Center, 3411 Wayne Avenue, Bronx, NY 10461 United States of America; 6 Department of Medicine (Endocrinology), Albert Einstein College of Medicine, 1300 Morris Park Avenue, Bronx, NY 10461 United States of America

**Keywords:** Health literacy, Mobile health, Type 2 diabetes, Socioeconomic factors, Diabetes Self-Management, eHealth literacy

## Abstract

**Purpose:**

Type 2 Diabetes Mellitus (T2DM) poses a growing public health burden, particularly among underserved populations. Mobile health (mHealth) apps offer a promising solution for improving self-management of T2DM, yet user engagement remains inconsistent across demographic and socioeconomic groups. This study aims to investigate the association between mobile health literacy, socioeconomic factors (including the Area Deprivation Index, ADI), and user engagement in the DiabetesXcel app, a digital diabetes management tool.

**Methods:**

A post-hoc analysis was conducted using data from 46 participants, and the analysis assessed app usage, clinical outcomes, and user characteristics. App engagement was measured by logins and in-app questions answered at four and six months.

**Results:**

Early users had significantly lower LDL levels after six months compared to sustained users (*p* = 0.02). Sustained users were more likely to have private insurance and less likely to have Medicaid (*p* = 0.04). Higher ADI was linked to increased eHealth literacy (*p* = 0.04) but lower social influence scores (*p* = 0.03). Non-private insurance users were less likely to answer more in-app questions at both four months (OR = 0.11, *p* = 0.05) and six months (OR = 0.14, *p* = 0.08), though these trends did not reach statistical significance.

**Conclusions:**

Disparities in socioeconomic status and digital literacy may influence sustained engagement with mHealth tools. Tailoring mHealth interventions to address disparities in engagement may improve long-term self-management behaviors in diverse patient populations.

**Supplementary Information:**

The online version contains supplementary material available at 10.1007/s40200-025-01725-2.

## Introduction

Type II Diabetes Mellitus (T2DM) is a widespread chronic metabolic disorder characterized by persistent hyperglycemia and insulin resistance. T2DM significantly increases an individual’s risk of cardiovascular disease, renal disease, amputations, and early-onset blindness, leading to considerable morbidity and mortality [1]. In 2022, the total cost of diagnosed diabetes in the United States was $412.9 billion, placing a significant strain on the healthcare system, with care for patients with diabetes accounting for one in four healthcare dollars and medical expenditures averaging 2.6 times more than those without diabetes [2].

Bronx County in New York City has the highest prevalence of diagnosed T2DM, affecting approximately 16% of adults [3]. The borough also experiences the highest rate of short-term diabetes-related hospitalizations statewide, at 6.8 per 10,000 residents—70% higher than the New York state average [4]. Marginalized populations in the Bronx face disproportionately higher rates of diabetes, compounded by reduced access to preventive care, including HbA1c testing, cholesterol screenings, and retinal exams [5, 6]. These disparities highlight the urgent need for innovative strategies to improve diabetes education, management, and self-care in underserved communities.

Managing chronic diseases like T2DM presents unique challenges, including limited access to specialty care, the need for lifestyle modifications, and gaps in provider training for culturally competent diabetes management. However, advancements in mobile health (mHealth) technology offer promising solutions to these barriers. The proliferation of smartphones has enabled the development of digital tools designed to enhance chronic disease self-management, particularly for patients in low-resource settings. In the past few years, mobile health (mHealth) applications (apps) have been increasingly effective in assisting patients with the self-management of chronic diseases like T2DM [5, 6]. With over 100,000 mHealth apps currently available on the Apple App Store (iOS) and Google Play Store (Android), this rapidly expanding market has the potential to reshape traditional healthcare by offering accessible and personalized disease management [7, 8]. Notably, research indicates that ethnically diverse and lower-income populations express greater interest in using mHealth apps for chronic disease management than their white, high-income counterparts [9–11]. This trend presents a unique opportunity to optimize mHealth interventions for communities like the Bronx, where digital tools could help bridge gaps in healthcare accessibility.

Among these technology-based innovations is DiabetesXcel, a mobile app designed to personalize and enhance diabetes self-management. DiabetesXcel offers guideline-based educational modules on blood glucose tracking, insulin dose recording, physical activity monitoring, and dietary logging, equipping users with evidence-based knowledge and practical insights to support informed decision-making [12]. Modeled after the successful ASTHMAXcel app, DiabetesXcel was tested in a single-arm trial with T2DM patients in the Bronx in 2021 [12]. Initial findings demonstrated improved diabetes outcomes, underscoring the need for further research to refine its features and maximize engagement [13].

Understanding demographic and socioeconomic factors influencing mHealth app usage is essential for optimizing user retention and long-term effectiveness. Existing research on mHealth engagement suggests that barriers to sustained use include lack of motivation, diminishing interest, and the preference for alternative apps [14]. Financial constraints are another key factor, as users often favor lower-cost options. However, these studies primarily focus on fitness and mental health apps, leaving a significant gap in research on mHealth engagement for chronic disease management. Moreover, retention rates for real-world mHealth applications remain low, with some studies reporting that fewer than 4% of users engage with health apps for more than 15 days [15]. These trends necessitate further investigation into the factors influencing long-term engagement, particularly for T2DM management.

The objective of this study was to analyze the demographic characteristics and app usage patterns of DiabetesXcel users. We examined factors such as age, gender, race/ethnicity, education, insurance status, socioeconomic environment based on geographic location, and insulin use to determine their association with app engagement. Based on previous literature, we hypothesized that younger users would engage with the app more frequently, while higher socioeconomic status (SES) and educational attainment would correlate with greater app usage [16, 17]. Given the limited research on demographic influences in chronic disease mHealth adoption, our study aims to bridge this gap and inform strategies for improving digital health interventions.

## Methods

### Study design

This study is a post-hoc analysis of data collected during a single-arm trial that evaluated the impact of the DiabetesXcel mobile app on diabetes self-management. The original study’s design and participant recruitment have been detailed previously [12]. In brief, participants were recruited from primary care and diabetes clinics across the Montefiore-Einstein Health System over a one-month period. Recruitment strategies included provider referrals and flyer distributions across clinic sites.

While the initial study assessed the app’s efficacy in improving diabetes-related outcomes, this post-hoc analysis focuses on how demographic factors and socioeconomic status (SES) influenced app engagement patterns. We examined baseline characteristics, including age, gender, race/ethnicity, education level, insurance type, insulin use, and Area Deprivation Index (ADI) scores, to determine their associations with app usage.

### Participants

Adults aged 18 years or older, English speaking, and diagnosed with T2DM were eligible for inclusion. Diagnosis criteria included a prior healthcare provider diagnosis, the use of glucose-lowering medications, or a hemoglobin A1c (HbA1c) level ≥ 6.5%. Participants had to be receiving diabetes care at Montefiore outpatient clinics and own a smartphone compatible with iOS or Android. Montefiore outpatient clinics were selected as the recruitment site due to their highly diverse underserved patient population, high prevalence of type 2 diabetes mellitus, and existing infrastructure to support digital mobile health research [18]. These clinics serve a large, socioeconomically varied community in the Bronx, NY, which is directly relevant to our study’s aim of examining the relationship between mobile health literacy, socioeconomic factors, and app engagement.

Exclusion criteria included pregnancy, major psychiatric or cognitive impairments, and chronic conditions requiring intensive treatment (e.g., chemotherapy, long-term steroid use). Participants lacking baseline data, such as initial HbA1c levels, LDL levels, or responses to the health literacy questionnaires, were excluded from this analysis, as these missing variables were essential for evaluating engagement and outcomes. Institutional Review Board approval was obtained from the Albert Einstein College of Medicine IRB Committee (#2018–9590), and all participants provided informed consent and HIPAA authorization.

### Mobile application

DiabetesXcel is an educational mHealth app designed to support diabetes self-management [19]. The app’s comprehensive design has been described by Berlot et al. [12]. DiabetesXcel is structured into nine chapters, each focused on educating patients about type 2 diabetes mellitus through animated videos. Topics include but are not limited to diabetes medications, diet, exercise, monitoring, and disease complications. Each chapter also includes six optional multiple-choice questions designed to reinforce the key points from the video content. All material was created per national diabetes guidelines, with input from diabetes experts [20]. The chapters mirror the curriculum of diabetes health educators’ sessions and were developed with input from both educators and endocrinology clinicians during the pre-development phase and were targeted at a 5th-grade reading level.

### Exposure variables

Data on participant characteristics were collected at baseline and included age, gender, race/ethnicity, education level, insurance type, insulin use, HbA1c, and low-density lipoprotein (LDL) levels. Race/ethnicity was dichotomized into Black or African American versus non-Black or African American. Education was classified as less than college graduate versus college graduate or higher, and insurance type was categorized as private versus non-private. These variables were analyzed to determine their impact on app engagement metrics.

### Data collection

Baseline characteristics were obtained through enrollment surveys, which gathered demographic information and medical history. Descriptions for the surveys are listed in Supplementary Table S1. In particular, the Social Influence (SI) Score, part of UTAUT questionnaire, assesses how social encouragement impacts a user’s adoption of a technology [21]. The eHealth Literacy Score (eHEALS) evaluates an individual’s ability to seek, understand, and apply online health information, both of which have been widely used in prior studies and in the ASTHMAXcel study to assess technology adoption and digital health engagement [22–25]. A complete list and description of all measures used in the study can be found in Supplementary Table S2.

Engagement with the DiabetesXcel app was monitored using integrated analytics, which recorded the number of logins, the number of application questions answered (AQA), and interactions with personalized push notifications. To further characterize engagement patterns, users were categorized as either early users or sustained users based on their login activity over time. Early users were defined as those who logged into the app within the first six months of the study. Sustained users were defined as those who continued logging into the app beyond the six-month mark. To characterize sustained use, we calculated the ratio of logins within the first six months to total logins recorded through November 2023, which was a post-study period well beyond the study’s conclusion. If this ratio equals 1, this indicates all logins occurred within the first 6 months, with no further engagement after the formal study window; these individuals are classified as early users. If the ratio is less than 1, this indicates additional logins occurred after the initial 6 months; these individuals are classified as sustained users. Further clarification can be found in Supplementary Note 1. These app usage metrics were collected at the four- and six-month time points to evaluate user engagement patterns throughout the study period, while login data was tracked beyond six months to assess long-term engagement. The Area Deprivation Index (ADI) was used to assess participants’ socioeconomic status, derived from their residential zip codes collected at baseline. The ADI is a composite index based on neighborhood-level factors such as income, education, and employment, influencing health outcomes. ADI was determined using the Neighborhood Atlas which maps participants’ zip codes to census tracts and generates deprivation scores. Higher ADI scores indicate greater socioeconomic deprivation. ADI can be calculated at different geographic levels, including State ADI and National ADI. State ADI is calculated using state-specific averages and provides a measure of socioeconomic deprivation relative to other areas within the same state. In contrast, national ADI is calculated using national averages, offering a broader measure of deprivation across the entire country. Both State ADI and National ADI were used in this study to assess participants’ socioeconomic status, allowing for comparisons at both local and national levels. All participant data and survey responses were securely managed through REDCap, while de-identified app usage data were stored on a secure cloud-based server dedicated to the DiabetesXcel app.

### Statistical analyses

We used logistic regression models to assess the impact of baseline participant characteristics on app engagement, defined by the number of logins and AQA at four and six months, which were our primary outcomes. Logins and AQA were dichotomized at the mean for each time point to account for skewed distributions. Odds ratios (OR) and 95% confidence intervals (CI) were calculated to evaluate the likelihood of answering more than the mean number of application questions or having more logins based on these characteristics. We used paired t-tests and chi-squared tests to compare demographic and clinical characteristics between early and sustained app users. Continuous variables were compared using means ± SD, while categorical variables were expressed as percentages. The ADI was analyzed using Pearson regression (linear correlation) to assess its correlation with app engagement and clinical outcomes.

The sample size varied slightly across analyses due to a small subset of participants missing data for specific variables. As a result, the sample was *n* = 46 at baseline, *n* = 44 for primary engagement analyses, and *n* = 43 for early vs. sustained user classification. Reasons for exclusion included missing baseline assessments, absence of app engagement data, and missing follow-up outcomes such as HbA1c or LDL. A detailed flow and missing data summary are provided in Supplementary Table S3. Statistical significance was set at *p* < 0.05, with trends considered at *p* < 0.10 to highlight potentially meaningful associations that may merit further investigation in larger studies. All analyses were conducted using STATA 17SE (College Station, TX) and SPSS (version 29, IBM, Chicago, IL).

## Results

### Baseline characteristics

A total of 55 individuals were recruited for the study. Of these, 50 completed the baseline assessment. Four participants were excluded for failing to register an account with DiabetesXcel or for not logging into the app, resulting in a final analytic sample of 46 participants.

Participants (*N* = 46) were on average 47 *±* 9 years old, mostly women (71.7%), with half identifying as Black or African American (50.0%) and having public insurance of Medicaid or Medicare (50.0%) (Table 1). Most of the participants reported educational attainment of less than a college degree. In terms of the severity of the participants’ diabetes, most reported a history of one or more T2DM-associated microvascular complications, including retinopathy, nephropathy, or neuropathy. Both iOS (Apple) and Android-based mobile devices were well represented in the cohort, with the slight majority (56%) of participants using the iOS-based software.

**Table 1:** Baseline characteristics of participants

	Total *N* = 46
Age (years, mean **±** SD)	47 *±* 9
Sex, n(%)	
Men	13 (28.26)
Women	33 (71.74)
Race/ethnicity, n(%)	
Black or African American	23 (50.00)
Hispanic	15 (32.61)
White or Caucasian	3 (6.52)
Other	5 (10.87)
Highest education received, n(%)	
Less than college grad or grad school	32 (69.57)
College graduate or graduate school	14 (30.43)
Insurance type, n(%)	
Private	22 (47.83)
Public (Medicaid or Medicare)	23 (50.00)
None	1 (2.17)
Type of diabetes complication, n(%)	
Microvascular, retinopathy, nephropathy, neuropathy	23 (79.31)
Cardiovascular disease	3 (10.34)
Foot damage	2 (6.90)
Skin conditions – fungal skin and nail infections	1 (3.45)
Medication, n(%)	
Metformin	22 (47.83)
Insulin	29 (63.04)
Oral anti-diabetes agent	5 (10.87)
Non-insulin injection	12 (26.09)
Other	9 (19.57)
Hemoglobin A1c (mg/dL)	8.7 *±* 2.0
Latest low-density lipoprotein (mg/dL)	123 *±* 40
Smartphone type, n(%)	
iOS	25 (55.56)
Android	20 (44.44)

### Application use and engagement patterns

The majority of logins occurred during the first 4 months after enrollment in the study. The average number of logins was 9 *±* 18 and AQA was 31 *±* 34 at 4 months. These figures increased marginally to 11 *±* 24 and 34 *±* 40 by the 6-month time point, respectively (Table 2). There were trends of older participants having greater odds of answering more than the mean AQA (OR = 1.09 [95% CI 0.99, 1.21], *p* = 0.07) at 6 months. Those of non-Black/African American race or ethnicity trended towards lower odds of answering more than the mean AQA at 6 months (OR = 0.14 [95% CI 0.02, 1.30], *p* = 0.08) compared to Black/African American participants, as did those with non-private insurance (OR = 0.14 [95% CI 0.02, 1.30], *p* = 0.08) compared to those with private insurance (Table 3). No other variables–including gender, insulin use, baseline hemoglobin A1c, and LDL levels– had a statistically significant association with the primary outcomes.

**Table 2:** Averages of application usage at 4 and 6 months

Outcome	4 months *N* = 44	6 months *N* = 44
	Mean *±* Standard deviation	
Logins	9 *±* 18	11 *±* 24
Application questions answered	31 *±* 34	34 *±* 40

**Table 3:** The association of baseline covariates with logins and number of application questions answered at 4 and 6 months. logins and application questions answered were dichotomized above and below the mean for each exposure and timepoint, respectively

	4-month logins *N* = 44	6-month logins *N* = 44	4-month application questions answered *N* = 44	6-month application questions answered *N* = 44
Baseline covariates				
Age	OR = 1.05 [95% CI 0.96, 1.15], *p* = 0.27	OR = 1.04 [95% CI 0.95, 1.15], *p* = 0.37	OR = 1.10 [95% CI 1.00, 1.21], *p* = 0.06	OR = 1.09 [95% CI 0.99, 1.21], *p* = 0.07
Gender				
Men	Reference	Reference	Reference	Reference
Women	OR = 0.93 [95% CI 0.15, 5.56], *p* = 0.93	OR = 0.62 [95% CI 0.10, 3.97], *p* = 0.61	OR = 0.87 [95% CI 0.15, 5.03], *p* = 0.87	OR = 0.45 [95% CI 0.05, 4.22], *p* = 0.48
Race/ethnicity				
Black or African American	Reference	Reference	Reference	Reference
Other	OR = 0.71 [95% CI 0.14, 3.63], *p* = 0.68	OR = 0.90 [95% CI 0.16, 5.04], *p* = 0.91	OR = 0.27 [95% CI 0.05, 1.50], *p* = 0.13	OR = 0.14 [95% CI 0.02, 1.30], *p* = 0.08
Education				
Less than college graduate or graduate school	Reference	Reference	Reference	Reference
College graduate or graduate school	OR = 0.83 [95% CI 0.14, 4.93], *p* = 0.84	OR = 1.23 [95% CI 0.20, 7.70], *p* = 0.83	OR = 0.38 [95% CI 0.08, 1.84], *p* = 0.23	OR = 0.49 [95% CI 0.09, 2.61], *p* = 0.41
Insulin use				
No	Reference	Reference	Reference	Reference
Yes	OR = 1.52 [95% CI 0.26, 8.93], *p* = 0.64	OR = 1.17 [95% CI 0.19, 7.21], *p* = 0.87	OR = 1.06 [95% CI 0.22, 5.18], *p* = 0.94	OR = 0.66 [95% CI 0.11, 3.86], *p* = 0.64
Insurance				
Private	Reference	Reference	Reference	Reference
Other	OR = 0.30 [95% CI 0.05, 1.78], *p* = 0.19	OR = 0.40 [95% CI 0.07, 2.48], *p* = 0.33	OR = 0.11 [95% CI 0.01, 1.03], *p* = 0.05	OR = 0.14 [95% CI 0.02, 1.30], *p* = 0.08
Hemoglobin A1c	OR = 1.06 [95% CI 0.72, 1.56], *p* = 0.78	OR = 1.13 [95% CI 0.76, 1.69], *p* = 0.55	OR = 0.86 [95% CI 0.60, 1.24], *p* = 0.42	OR = 0.87 [95% CI 0.60, 1.27], *p* = 0.48
Low-density lipoprotein	OR = 1.01 [95% CI 0.99, 1.03], *p* = 0.59	OR = 1.01 [95% CI 0.99, 1.03], *p* = 0.34	OR = 1.00 [95% CI 0.98, 1.02], *p* = 0.80	OR = 1.00 [95% CI 0.98, 1.02], *p* = 0.77

Furthermore, an analysis of usage patterns revealed that early users (*n* = 34) experienced a greater reduction in LDL levels compared to sustained users (*n* = 9); this difference in LDL values between the groups at post-study follow-up was significant (*p* = 0.02). After six months of app use, the mean change in LDL for early users was − 32.06 *±* 19.05, whereas sustained users experienced a mean change of −15.67 *±* 16.65. Additionally, sustained users were more likely to have private insurance (7 *±* 77.8) and less likely to have Medicaid (0 *±* 0) (*p* = 0.04), emphasizing the socioeconomic differences associated with app engagement (Table 4).

**Table 4:** Characteristics of early and sustained users (*N* = 43)

	Early (*n* = 34)	Sustained (*n* = 9)	*p*-value
Age (years, mean ± SD)	47.71 ± 8.53	47.56 ± 10.60	0.96
Sex			0.72
Male	7 ± 20.6	3 ± 33.3	
Female	27 ± 79.4	6 ± 66.7	
Race/Ethnicity			0.61
Black or AA	15 ± 44.1	6 ± 66.7	
Hispanic	12 ± 35.3	2 ± 22.2	
White/Caucasian	2 ± 5.9	1 ± 11.1	
Asian	2 ± 5.9	0 ± 0.0	
Other	3 ± 8.8	0 ± 0.0	
State Deprivation Index (mean ± SD)	5.41 ± 1.67	5.11 ± 2.37	0.66
National Deprivation Index (mean ± SD)	26.00 ± 17.62	27.78 ± 28.11	0.81
Change in A1C (mean ± SD)	−1.08 ± 1.01	−1.09 ± 0.59	0.99
Change in LDL (mean ± SD)	−32.06 ± 19.05	−15.67 ± 16.65	0.02
Type of Insurance (%)			0.04
Medicaid	17 ± 50.0	0 ± 0.0	
Medicare	3 ± 8.8	2 ± 22.2	
Private	13 ± 38.2	7 ± 77.8	
No Insurance	1 ± 2.9	0 ± 0.0	
Highest Education Level (%)			0.49
Less than High School	2 ± 5.9	0 ± 0.0	
Some High School	3 ± 8.8	0 ± 0.0	
GED	0 ± 0.0	1 ± 11.1	
High School Graduate	10 ± 29.4	3 ± 33.	
Tech School/Some College	9 ± 26.5	3 ± 33.3	
College Graduate	6 ± 17.6	1 ± 11.1	
Graduate School	4 ± 11.8	1 ± 11.1	

### ADI and eHealth literacy

Pearson’s correlation coefficients (r) showed that e-health literacy scores were positively associated with National ADI (*r* = 0.30, *p* = 0.04), while social influence scores were inversely correlated with National ADI (*r* = −0.33, *p* = 0.03) (Table 5). No other variables, including A1c change or LDL change, had a statistically significant association with ADI.

**Table 5:** Pearson’s correlation coefficient (r) between area deprivation index and key outcome variables

	StateADI	*p*-value	National ADI	*p*-value
A1C Change	−0.15	0.33	−0.05	0.74
LDL Change	−0.18	0.25	−0.05	0.76
eHealth Literacy Score	0.36	0.01	0.30	0.04
Social Influence Score	−0.23	0.12	−0.33	0.03

*Note: Values represent Pearson’s correlation coefficients (r), measuring the linear association between State/National ADI and each outcome (A1c Change, LDL Change, eHealth Literacy Score, and Social Influence Score)

## Discussion

Our study cohort was predominantly female and racially diverse, with half of participants identifying as Black/African American and half insured through Medicaid or Medicare. The high proportion of publicly insured individuals highlights the socioeconomic vulnerabilities within our study population, which may contribute to disparities in healthcare access and chronic disease management. Additionally, the high prevalence of microvascular complications suggests that many participants had advanced diabetes, reinforcing the need for effective self-management interventions in this high-risk population.

In contrast to prior research linking lower socioeconomic status with decreased engagement in digital health tools due to barriers such as limited technology access, lower digital literacy, and diminished health literacy [26–30], findings from the DiabetesXcel app show that participants from socioeconomically disadvantaged areas reported higher e-health literacy. This suggests that digital health interventions can effectively reach underserved populations and highlights the complexity of the relationship between resource deprivation and digital literacy. One explanation is the tailored design of DiabetesXcel, which was created at a 5th-grade reading level with engaging educational materials to reduce literacy barriers. The app’s personalized features, such as language and content customization, may have further contributed to higher engagement. A study on mHealth apps for diverse, low-income populations found that simplified, patient-friendly language with minimal medical jargon improved usability and engagement [31]. This may reflect an increased reliance on digital tools to compensate for limited healthcare access, emphasizing the potential of well-designed mHealth interventions in mitigating health disparities [31].

Notably, early users of the app experienced a significantly greater reduction in LDL levels compared to sustained users. This suggests that while the initial six-month period of app use was effective in improving lipid levels, continued use did not yield comparable benefits. This finding may be attributed to changes in user behavior, engagement patterns, or diminishing intervention effects over time. Research indicates that user retention in health apps tends to decline, with many users discontinuing after achieving their desired health outcomes [31]. Additionally, prior literature suggests that health improvements from mHealth interventions may follow a plateau effect, where early engagement yields the most substantial benefit, and continued use offers diminishing returns. The concept of “app fatigue” may also play a role, as engagement in digital health tools frequently declines after an initial period of motivation [32]. A systematic review found that over 50% of users disengage within the first month, reinforcing the need for retention strategies such as gamification and personalized feedback [33, 34]. Future interventions should incorporate strategies to sustain engagement, including updated content, behavioral incentives, and targeted user support. Gamification is one potential strategy to enhance engagement, as prior studies suggest that elements like digital rewards, leaderboards, and progress tracking can increase motivation [35–38]. While our study did not directly assess gamification effects, future research should explore whether such features influence long-term engagement in mHealth interventions in patients with chronic disease.

Despite high engagement among socioeconomically disadvantaged individuals, our results showed a significant association between sustained engagement and insurance status. Specifically, sustained users were more likely to have private insurance and less likely to have Medicaid. This suggests that individuals with private insurance may have more access to healthcare resources, facilitating long-term engagement with the app. In contrast, Medicaid users may encounter barriers such as limited internet access, fewer healthcare visits, and financial instability, which can hinder their ability to consistently engage with digital health tools [39, 40]. Prior studies have reported that higher income and better education levels are associated with increased interest in and utilization of digital health services [41]. Additionally, studies suggest that individuals with Medicaid may have competing social and health-related stressors that limit sustained engagement with self-management tools [42, 43]. These disparities highlight the need to design digital health interventions that accommodate structural barriers faced by low-income users and ensure equitable access.

Our study also revealed an unexpected positive association between e-health literacy and the Area Deprivation Index (ADI), suggesting that participants from more socioeconomically deprived areas reported higher levels of e-health literacy. These finding challenges traditional assumptions that lower socioeconomic status is uniformly associated with decreased digital health competency. One possible explanation is that individuals in resource-limited settings may develop digital proficiency out of necessity, particularly as mobile health (mHealth) tools become a primary means of accessing healthcare resources [40]. However, despite higher e-health literacy, our results also indicated that sustained engagement with the DiabetesXcel app was significantly associated with private insurance status, while those with Medicaid were less likely to continue long-term app usage. This suggests that structural barriers—such as inconsistent internet access, financial instability, and reduced healthcare support—may still impede engagement, despite digital literacy [41]. Additionally, we observed an inverse correlation between social influence scores and ADI, indicating that participants in more socioeconomically disadvantaged areas reported lower levels of social support related to diabetes management. Social support plays a crucial role in sustaining health behavior changes, and its absence may contribute to lower long-term engagement with mHealth interventions [44]. Prior research highlights that individuals in low socioeconomic conditions often experience social isolation and reduced community resources, which can further limit their ability to fully integrate digital health tools into their self-management routines [45]. These findings underscore the need for digital health strategies that not only enhance accessibility but also address broader social determinants of health, ensuring equitable engagement for populations facing socioeconomic barriers.

Several limitations must be acknowledged. The single-arm observational design restricts our ability to determine causality, and without a control group, we cannot definitively attribute observed outcomes solely to the DiabetesXcel app. Additionally, participants who did not log in were excluded, which may introduce selection bias and affect the generalizability of our findings. Another limitation is that the app was available only in English, potentially limiting accessibility for non-English speakers, particularly in a diverse area like the Bronx, NY. The limited sample size restricted statistical power and prevented some findings from reaching significance. However, the observed trends suggest meaningful patterns that warrant further investigation and post hoc analysis. Furthermore, our analysis relied on self-reported engagement data, which may be subject to recall bias. While the study included a follow-up period, longer-term trends in engagement and clinical outcomes remain unclear. Future studies should explore controlled trial designs, multilingual support, and objective engagement tracking to address these limitations.

In addition to these limitations, there are potential confounding factors that may have influenced both app engagement and clinical outcomes [46]. For example, individuals with greater intrinsic motivation or self-efficacy in managing chronic illness may have been more likely to engage with the app consistently, potentially inflating the app’s apparent effectiveness. Similarly, provider encouragement, whether through direct recommendation or ongoing reinforcement, could have enhanced both participant engagement and adherence to self-care behaviors. Unmeasured comorbidities, such as depression or other psychosocial stressors, may have undermined participants’ capacity to use the app regularly or act on its content. Moreover, broader social determinants of health, including housing instability, employment status, caregiving status, and access to reliable internet, likely influenced participants’ ability to engage with the digital platform. Future research could aim to incorporate more comprehensive measures of individual, clinical, and contextual variables to better understand the mechanisms driving digital health engagement and outcomes.

In summary, the DiabetesXcel app demonstrated strong engagement, particularly among socioeconomically disadvantaged users, challenging prior assumptions about digital literacy barriers. However, engagement disparities based on insurance status and the observed attrition rates highlight the need for further investigation into long-term retention strategies. Future research should explore ways to enhance sustained engagement, including content updates, gamification elements, and targeted user support. Additionally, investigating subgroup-specific engagement trends, such as differences by age, gender, or comorbidity burden, may help refine mHealth interventions for more personalized chronic disease management. Understanding these factors will be critical in optimizing mHealth interventions for chronic disease management, particularly among populations facing healthcare access barriers [47–49].

Building on prior research, our study challenges conventional assumptions about digital health engagement by demonstrating that participants from socioeconomically deprived areas reported higher levels of e-health literacy. This finding suggests that digital health interventions may serve as a crucial resource in communities with limited healthcare access. Additionally, we observed trends indicating that older adults engaged more with the DiabetesXcel app, while those identifying as non-Black/African American and individuals with non-private insurance were less likely to engage. Notably, sustained app users were more likely to have private insurance, underscoring the complex interplay between socioeconomic status and long-term digital health adoption.

Despite these promising engagement trends, attrition rates remained high, reinforcing the broader challenge of long-term retention in mHealth applications. Moreover, targeted adaptations for underrepresented groups—such as individuals with public insurance or lower initial engagement—may help bridge gaps in digital health accessibility and effectiveness. Ultimately, further research is essential to explore these relationships in depth and develop tailored strategies to maximize the impact of mHealth tools in chronic disease management, particularly for underserved populations.

## Supplementary Information

Below is the link to the electronic supplementary material.

Supplementary Material 1 (DOCX 23.0 KB)

## Data Availability

The datasets generated and/or analyzed during the current study are not publicly available due to privacy and ethical restrictions, but are available from the corresponding author on reasonable request.

## Abbreviations

(T2DM): Type 2 Diabetes Mellitus
(HbA1c): Hemoglobin A1c
(LDL): Low Density Lipoprotein
(mHealth): Mobile Health
(ADI): Area Deprivation Index
(DKQ-24): Diabetes Knowledge Questionnaire-24
(DSES): Diabetes Self-Efficacy Scale
(DSMQ): Diabetes Self-Management Questionnaire
(PHQ-9): Patient Health Questionnaire
(eHEALS): eHealth Literacy Scale
(NVS): Newest Vital Sign
(OR): Odds Ratio
(CI): Confidence Interval
(QoL): Quality of Life Score
(BMI): Body Mass Index
(HIPAA): Health Insurance Portability and Accountability Act
(GED): General Educational Development
(SI): Social Influence Score
(FC): Facilitating Conditions Score
(UTAUT): Unified Theory of Acceptance and Use of Technology Score
