# Supplementary material for: The Impact of Mobile Health Literacy, Socioeconomic Factors, and Engagement Patterns on DiabetesXcel App Usage in Adults
Source: J Diabetes Metab Disord. 2025 Sep 25;24(2):210. doi: 10.1007/s40200-025-01725-2 (PMC12463775; doi:10.1007/s40200-025-01725-2)
Supplement: Supplementary file 1 — Supplementary Material 1 (DOCX 23.0 KB) [file 40200_2025_1725_MOESM1_ESM.docx]

**Supplemental Table S1:** Comparison of App Engagement, eHealth Literacy, and Diabetes-Related Scores Between Early and Sustained Users

|  | Early (n = 34) | Sustained (n = 9) | p-value |
| --- | --- | --- | --- |
| eHealth Literacy Score (eHeals Score) (mean ± SD) | 30.83 **±** 5.36 | 31 **±** 4.24 | 0.97 |
| Screening Score (mean ± SD) | 28.71 **±** 6.5 | 30.44 **±** 3.28 | 0.28 |
| Terminology and System Information Score (mean ± SD) | 39.68 **±** 9.14 | 38.44 **±** 17.37 | 0.84 |
| Learning Score (mean ± SD) | 39.15 **±** 10.2 | 36.22 **±** 18.40 | 0.66 |
| System Capability Score (mean ± SD) | 20.03 **±** 6.34 | 19.89 **±** 8.52 | 0.96 |
| User Interface Score (mean ± SD) | 31.68 **±** 9.99 | 33.22 **±** 14.02 | 0.76 |
| Total Questionnaire for User Interface Satisfaction Score (mean ± SD) | 189.03 **±** 41.88 | 185.44 **±** 70.74 | 0.89 |
| Performance Expectancy Total Score (mean ± SD) | 13.76 **±** 3.6 | 13.78 **±** 2.77 | 0.99 |
| Effort Expectancy Total Score (mean ± SD) | 16.85 **±** 2.68 | 16.00 **±** 2.83 | 0.43 |
| Social Influence Total Score (mean ± SD) | 13.88 **±** 3.82 | 15.22 **±** 2.05 | 0.17 |
| Facilitating Conditions Total Score (mean ± SD) | 16.71 **±** 2.67 | 17.22 **±** 2.11 | 0.55 |
| Unified Theory of Acceptance and use of Technology Total Score (mean ± SD) | 61.21 **±** 10.50 | 62.22 **±** 6.96 | 0.73 |
| Satisfaction Total Score (mean ± SD) | 23.32 **±** 5.81 | 25.78 **±** 6.70 | 0.34 |
| Impact Total Score (mean ± SD) | 15.56 **±** 5.02 | 15.67 **±** 4.2 | 0.95 |
| Worry Total Score (mean ± SD) | 10.21 **±** 7.9 | 9.78 **±** 10.64 | 0.91 |
| Total Diabetes Quality of Life Score (mean ± SD) | 49.09 **±** 15.11 | 51.22 **±** 19.09 | 0.76 |
| Total Diabetes Self-Management Questionnaire Score (mean ± SD) | 21.94 **±** 5.67 | 21.11 **±** 3.41 | 0.58 |
| Total Diabetes Knowledge Questionnaire Score (mean ± SD) | 17.09 **±** 2.82 | 16.78 **±** 2.82 | 0.77 |
| Total Diabetes Self-Efficacy (mean ± SD) | 50.76 **±** 14.16 | 51.78 **±** 10.21 | 0.81 |
| Total Score (mean ± SD) | 4.85 **±** 5.80 | 5.00 **±** 7.66 | 0.96 |
| Patient Health Questionnaire Total Score (mean ± SD) | 25.97 **±** 4.58 | 26.56 **±** 3.54 | 0.69 |

**Supplemental Table S2:** Description of Measures Used in the Study

| **Measure** | **Description** |
| --- | --- |
| eHealth Literacy Score (eHEALS) | Measures a person's ability to effectively locate, evaluate, and use electronic health resources. |
| Screening Score | Evaluates screen-related usability aspects, such as readability, organization, and navigation clarity. |
| Terminology and System Information Score (TSI) | Assesses the clarity, consistency, and relevance of terminology and system messages within the diabetes management application. |
| Learning Score | Reflects how easy it is to learn and remember how to use the application. |
| System Capability Score | Evaluates how well the system responds to user input, corrects mistakes, and accommodates different user experience levels. |
| User Interface Score (UI) | Focuses on visual elements, including color, feedback, error handling, and overall interface design. |
| Total Questionnaire for User Interface Satisfaction Score (QUIS) | A comprehensive measure of user satisfaction, aggregating multiple usability sub-scores. |
| Performance Expectancy Total Score (PE) | Captures how much users believe the app helps them manage their diabetes effectively (e.g., taking medication, tracking symptoms). |
| Effort Expectancy Total Score (EE) | Measures how easy the app is to use and learn. |
| Social Influence Total Score (SI) | Evaluates external encouragement to use the app, such as support from family, friends, or healthcare providers. |
| Facilitating Conditions Total Score (FC) | Assesses whether users have the resources and knowledge needed to use the app effectively. |
| Unified Theory of Acceptance and Use of Technology Total Score (UTAUT) | A total score incorporating PE, EE, SI, and FC to measure technology adoption. |
| Satisfaction Total Score | Measures how content users are with different aspects of diabetes management and their daily routine. |
| Impact Total Score | Reflects how much diabetes interferes with daily life, including social interactions and driving. |
| Worry Total Score | Captures concerns related to diabetes, such as employment, relationships, and future health risks. |
| Diabetes Quality of Life Score (DQOL) | Evaluates the overall impact of diabetes on life satisfaction. |
| Diabetes Self-Management Questionnaire Score (DSMQ) | Measures self-care behaviors like medication adherence, diet, and exercise. |
| Diabetes Knowledge Questionnaire Score (DKQ) | Assesses knowledge of diabetes management principles. |
| Diabetes Self-Efficacy Score | Evaluates confidence in managing diabetes independently. |
| Total Score | A summary score aggregating various health and usability metrics. |
| Patient Health Questionnaire Total Score (PHQ) | Assesses mental health status, particularly depression severity. |

**Supplemental Table S3:** Participants Inclusion and Exclusion at Key Analytical Stages

| **Stage** | **n** | **Reason for Exclusion** |
| --- | --- | --- |
| Recruited | 55 |  |
| Completed baseline assessment | 50 | 5 did not complete baseline surveys |
| Registered and used the app | 46 | 4 did not register for or use the app |
| Engagement analysis (4 and 6 months) | 44 | 2 missing follow-up/app usage data |
| Early/sustained user classification | 43 | 1 did not log in during initial 6-month window |

**Supplementary Note 1: Definition of Sustained Use Ratio**

**Sustained Use Ratio = (Number of logins within first 6 months) / (Total logins through November 2023)**

- **Definition and Classification:**
  - The numerator represents all logins that occurred during the defined 6-month study period.
  - The denominator is the total number of logins recorded from enrollment through the post-study follow-up period, ending in November 2023 (which included additional logins beyond the formal study window).
  - If the ratio equals **1**, this indicates all logins occurred within the first 6 months, with no further engagement after the formal study window; these individuals are classified as **early users**.
  - If the ratio is **less than 1**, this indicates additional logins occurred after the initial 6 months; these individuals are classified as **sustained users**.
- **Worked Examples:**
  - *Example 1*: A participant logs in 8 times in the first 6 months and never again; their sustained use ratio is 8/8 = 1 (**early user**).
  - *Example 2*: A participant logs in 8 times in the first 6 months and then 4 more times during the follow-up period; their sustained use ratio is 8/12 ≈ 0.67 (**sustained user**).
- **Interpretation:**
  The lower the ratio, the greater the proportion of post-study engagement, reflecting long-term app use.
